# Genetic Polymorphism of Zinc Transporter-8 Gene (SLC30A8), Serum Zinc Concentrations, and Proteome Profiles Related to Type 2 Diabetes in Elderly

**DOI:** 10.3390/jcm14030790

**Published:** 2025-01-25

**Authors:** Jintana Sirivarasai, Pimvaree Tristitworn, Prapimporn Chattranukulchai Shantavasinkul, Sittiruk Roytrakul, Sirintorn Chansirikarnjana, Sirasa Ruangritchankul, Suwannee Chanprasertyothin, Piangporn Charernwat, Pachara Panpunuan, Thanyachai Sura, Piyamitr Sritara

**Affiliations:** 1 Nutrition Unit, Faculty of Medicine Ramathibodi Hospital, Mahidol University, Bangkok 10400, Thailand; 2 Master of Science Program in Nutrition, Faculty of Medicine Ramathibodi Hospital and Institute of Nutrition, Mahidol University, Bangkok 10400, Thailand; buay.pmv@gmail.com; 3 Department of Medicine, Faculty of Medicine Ramathibodi Hospital, Mahidol University, Bangkok 10400, Thailand; sprapimporn@gmail.com (P.C.S.); chansirikarn.s@gmail.com (S.C.); sirasarama37@gmail.com (S.R.); piangpornc@gmail.com (P.C.); pachpan@hotmail.com (P.P.); thanyachai.sur@mahidol.ac.th (T.S.); piyamitr.sri@mahidol.ac.th (P.S.); 4 National Center for Genetic Engineering and Biotechnology, 113 Thailand Science Park, Pathum Thani 12120, Thailand; sittiruk@biotec.or.th; 5 Research & Innovation, Faculty of Medicine Ramathibodi Hospital, Mahidol University, Bangkok 10400, Thailand; suwannee.cha@mahidol.ac.th

**Keywords:** zinc transporter, genetic polymorphism, proteomics, type 2 diabetes, elderly

## Abstract

**Background and Aims**: Older adults are particularly susceptible to type 2 diabetes mellitus (T2DM) due to factors such as age-related insulin resistance, decreased physical activity, and deficiency of micronutrients, especially zinc. Studies have suggested that the risk allele of the zinc transporter 8 gene (SLC30A8) single-nucleotide poly-morphism (SNP) rs13266634 may contribute to T2DM susceptibility in addition to the complex protein interactions and alterations in the protein expressions and modifications associated with T2DM. This study was implemented to study the associations between SLC30A8 polymorphism, serum zinc levels, and the profiles of proteins differentially expressed in nondiabetic (*n* = 116) and prediabetic/diabetic (*n* = 149) subjects. **Methods**: SNP genotyping using TaqMan^®^ assay and proteomic analysis by LC-MS/MS were performed in each group. **Results**: The results showed a higher risk of diabetes in individuals with the risk genotype CC accompanied by a low serum zinc level than in those with other genotypes. Profiles of proteins differentially expressed between the groups were identified and shown to be particularly associated with zinc-related functions, zinc transporter 8, and glucose metabolism. Proteins exclusively expressed in prediabetes/diabetes were assigned to a Reactome pathway related to zinc transporter and insulin processing. **Conclusions**: Our findings suggest that individuals carrying at least one copy of SLC30A8 rs13266634 accompanied by a low serum zinc level might be susceptible to T2DM, which could be due to alterations in insulin signaling and zinc metabolism. Understanding this relationship deepens our understanding of the genetic and molecular mechanisms underlying T2DM risk, offering potential targets for therapeutic intervention and prevention strategies.

## 1. Introduction

Zinc is an essential trace element that plays particularly significant roles in antioxidant enzymes and is crucial for many aspects of metabolism, including gene expression, enzymatic reactions, protein synthesis, DNA synthesis, wound healing, and growth and development [1,2]. It has also been proposed that zinc is involved in the etiology of type 2 diabetes mellitus (T2DM) due to its significant role in glucose metabolism, especially in the synthesis, storage, and secretion of insulin as well as the conformational integrity of insulin in its hexameric form [3]. Given these integral functions, a decrease in zinc level can lead to pancreatic islet β-cell dysfunction and insulin resistance, which are considered two major factors in T2DM pathogenesis [3]. Older people are particularly susceptible to zinc deficiency due to factors such as reduced dietary intake, impaired absorption, chronic health conditions, and the use of medications that can affect zinc metabolism [4]. The involvement of zinc in T2DM can also be explained by the crucial roles it plays in many signaling pathways, such as the phosphoinositide 3-kinase/protein kinase B signaling pathway, which promotes glucose uptake in peripheral tissues [5], and the AMP-activated protein kinase pathway, which regulates the energy balance relative to insulin sensitivity and prevents β-cell dysfunction [6].

A previous study in diabetic patients and nondiabetic controls investigated zinc levels and their association with glycemic parameters and insulin and glucagon levels. The results showed a significant decrease in serum zinc levels in T2DM patients compared with those of the controls (*p* = 0.02). Patients with lower zinc levels exhibited higher fasting insulin levels (*p* = 0.006) and a higher β-cell activity index (*p* = 0.02). Notably, there were no significant differences in fasting glucagon, fasting blood glucose (FBG), 2 h postprandial blood glucose, and glycated hemoglobin (HbA1C) between the two groups [7]. Another study of 150 patients with T2DM and 50 controls showed that zinc levels were significantly lower in the T2DM group (62.89 µg/dL) than in the controls (74.95 µg/dL, *p* < 0.05). Moreover, results from a multivariate analysis and systematic review indicated a significant link between zinc levels and the prevalence of T2DM [8,9].

Zinc transporter 8 (ZnT-8), a member of the zinc transporter family, is encoded by the solute carrier family 30 member 8 gene (SLC30A8) on chromosome 8q24.11 and expressed predominantly in pancreatic β cells [10]. The main function of ZnT-8 is to transport zinc from the cytoplasm into insulin secretory vesicles, in which insulin is stored in a solid hexamer bound with two Zn2+ ions before its secretion [11]. Its importance is highlighted by the finding that variants of this gene can lead to susceptibility to developing T2DM. Data from several genome-wide association studies have identified genetic variants that increase the risk of T2DM in humans, regarding the increased risk of T2DM conferred by the C allele of rs13266634 [12,13]. Shan et al. conducted a case–control study to explore how SLC30A8 interacts with plasma zinc levels in relation to T2DM. They found that while the C allele of the rs13266634 variant increases the risk of T2DM, higher plasma zinc levels decrease this risk and plasma zinc could be influenced by the SLC30A8 rs13266634 variant [14]. Another case–control study that enrolled 358 T2DM patients and 326 healthy controls revealed a significant association between the C/T rs13266634 SNP and T2DM in the Jordanian population [15]. Specifically, compared with those having the T allele, individuals with the C allele had a higher risk of T2DM (OR = 1.47; 95% CI: 1.14–1.89; *p* = 0.003), suggesting that rs13266634 is a strong candidate marker for identifying those at risk of developing T2DM [15]. Additionally, a systematic review and meta-analysis reported an association between various SNPs and the development of obesity and T2DM in Asian populations, with strong associations for T2DM at an odds ratio (OR) of 1.22 for SNP rs13266634 [16].

Proteomics refers to the large-scale study of proteins, focusing on their functions and expression. The proteomic analysis of T2DM in this research is intended to improve our understanding of the disease mechanisms, identify biomarkers for early diagnosis, and help to develop targeted therapies. By analyzing serum proteomic profiles, changes in the protein expression associated with insulin resistance, β-cell dysfunction, and systemic inflammation frequently observed in T2DM can be revealed [17]. A comparative protein expression analysis between healthy subjects without metabolic syndrome (*n* = 60) and those recently diagnosed with T2DM (*n* = 87) showed that 90 proteins were significantly dysregulated in the two groups, including 32 proteins that had not previously been associated with T2DM; among the identified proteins was S100A6, which was suggested to play a critical role in the pathogenesis of T2DM [18]. Another previous study examined the proteomic profiles in two large longitudinal cohorts (*n* = 2839). The results indicated that ACY1 was strongly associated with the risk of T2DM. This protein may influence amino acid metabolism and insulin homeostasis both in vitro and in vivo [19]. In another study, the plasma proteome related to dietary zinc depletion and repletion was analyzed using MALDI-TOF mass spectrometry. This work identified two key proteins: fibrin β, potentially linked to increases in liver fibrin induced by zinc deficiency, and the fibrin component chain E, fragment double D chain E, possibly the result of changes in the activity of thrombin-activatable fibrinolysis inhibitor [20]. Elsewhere, a multiomics analysis of zinc-deficient rats using serum proteomics, metabolomics, and liver proteomics identified potential biomarkers of zinc deficiency and highlighted its negative effects. Moreover, glutathione sulfotransferase omega-1 was found to show a strong response to zinc supplementation, suggesting its potential as a diagnostic biomarker for zinc deficiency [21].

Despite the above findings, there is limited data on the influence of genetic variations in the ZnT family, serum zinc levels, and protein expression and function on T2DM. A novel contribution of this study might involve investigating the specific effects of low serum zinc levels on T2D, potentially focusing on zinc homeostasis regulation, pancreatic islet function, or interactions between zinc proteins and other biomolecules. By examining the proteomic changes associated with low serum zinc levels, this study could provide valuable insights into the molecular mechanisms underpinning the link between zinc deficiency and T2D, ultimately contributing to the development of targeted therapeutic strategies or preventive measures for high-risk populations.

## 2. Materials and Methods

### 2.1. Study Population

This cross-sectional study, part of the Electricity Generating Authority of Thailand study (EGAT) cohort study, focused on 265 older Thai individuals aged 60–80 years. Comprehensive data on these individuals were collected in 2012, with all participants completing a self-administered sociodemographic questionnaire. Exclusion criteria for this study were as follows: acute infections, acute cardiovascular diseases, active liver disease or dysfunction, renal impairment, and overt hematological or malignant diseases. Subjects who were on insulin or taking any antioxidant or zinc supplements were also excluded.

At the time of enrollment, the physician performed a physical examination and anthropometric measurements for each subject, and blood samples were collected. Each participant provided written informed consent, and the protocol was approved by the Ethical Clearance Committee on Human Rights Related to Research Involving Human Subjects, Faculty of Medicine Ramathibodi Hospital, Mahidol University (COA.MURA2020/1790, 12 November 2020).

Anthropometric measurements, including waist and hip circumferences, were made using a flexible nonelastic measuring tape, with individuals standing with their feet together and arms resting at their sides. The hip circumference was measured as the maximum perimeter of the buttocks, while the waist circumference (WC) was measured at the plane between the navel and the inferior rib border. For this study, we used the 1998 WHO definitions of “overweight” and “central adiposity” as body mass index (BMI, in kg/m^2^) ≥ 25 and WC ≥ 94 cm (Asian ≥ 90 cm) for men and ≥ 80 cm for women [22].

We also applied the diagnostic criteria established by the American Diabetes Association as follows: Prediabetes was defined by either an HbA1C level of 5.7–6.4% or a fasting plasma glucose (FPG) level of 100 mg/dL (5.6 mmol/L) to 125 mg/dL. T2DM was defined by an FPG level higher than 126 mg/dL (7.0 mmol/L) or an HbA1C level higher than 6.5% (48 mmol/mol) [23]. Meanwhile, in accordance with the NCEP ATP III definition, metabolic syndrome was diagnosed when three or more of the following five criteria were met: (1) WC greater than 40 cm in men or 35 cm in women, (2) blood pressure exceeding 130/85 mmHg, (3) fasting triglyceride (TG) level greater than 150 mg/dL, (4) fasting high-density lipoprotein cholesterol (HDL-C) level less than 40 mg/dL in men or 50 mg/dL in women, and (5) fasting plasma glucose level over 100 mg/dL [24].

### 2.2. Biochemical Analysis

A 6 mL blood sample was carefully collected from each subject after fasting overnight for 8–12 h. Subsequently, serum and plasma samples were separated from the whole blood by centrifugation at 3000× *g* for 10 min. The metabolic profile of the subjects was then evaluated, focusing on various key parameters including glucose, total cholesterol (TC), HDL-C, low-density lipoprotein cholesterol (LDL-C), blood urea nitrogen, creatinine, uric acid, albumin, aspartate aminotransferase (AST), alanine aminotransferase (ALT), high-sensitivity C-reactive protein (hsCRP), and homocysteine (Hcy) levels. These measurements were carried out using conventional methods on advanced laboratory equipment, specifically the Cobas Analyzer and the Cobas Integra Analyzer (Roche Diagnostics Ltd., Basel, Switzerland).

Analysis of serum zinc was performed using an inductively coupled plasma mass spectrometer (ICP-MS), specifically the Agilent 7700× ICP-MS (Agilent Technologies, Santa Clara, CA, USA), following a modified version of the method reported by Krachle et al. [25].

### 2.3. Genotyping

Genomic DNA was successfully isolated from 3 mL of peripheral blood collected in EDTA tubes using the “salting-out” method [26]. To genotype the subjects in terms of the SLC30A8 SNP (rs13266634), we used the TaqMan method (Applied Biosystems, Foster City, CA, USA). The primers and probes for TaqMan SNP genotyping were expertly designed by Applied Biosystems, with assay ID C____357888_10 and context sequence [VIC/FAM:TGCTTCTTTATCAACAGCAGCCAGC[C/T]GGGACAGCCAAGTGGTT CGG AGAGA]. Oligonucleotides were synthesized with FAM and VIC fluorogenic markers attached to the 5′ ends of the probes, facilitating precise allelic discrimination. The assays were carried out in accordance with the manufacturer’s protocol, incorporating 2 µL of DNA in a total reaction volume of 5 µL in 96-well plates. The TaqMan real-time polymerase chain reaction assays were performed with the following program: 95 °C for 10 min, followed by 40 cycles of 95 °C for 15 s and 60 °C for 1 min, in strict adherence to the manufacturer’s instructions.

### 2.4. Proteomic Analysis

A proteomic analysis was performed using LC-MS/MS, as described previously [27]. A total of 20 µg of pooled serum protein was obtained from 116 subjects in the non-diabetes group and 149 in the prediabetes/diabetes group. Total protein was extracted, protein content was evaluated using the Lowry method, and in-gel digestion was performed as described previously [28]. In this study, we employed MaxQuant 1.6.1.12, a widely used software platform for quantitative proteomics that was specifically designed for analyzing mass spectrometry data. This software features an integrated search engine, typically based on the Andromeda algorithm, and provides comprehensive tools for statistical analysis, visualization, and data interpretation. For processing the MS data, the following parameters were applied: a maximum of two missed cleavages, mass tolerance of 20 ppm for the main search, trypsin as the digesting enzyme, and carbamidomethylation of cysteines as a fixed modification. Additionally, oxidation of methionine and acetylation of the protein N-terminus were considered variable modifications. Peptides were required to have a minimum length of seven amino acids and at least one unique peptide to qualify for protein identification. Proteins were considered identified only if they had at least two peptides and one unique peptide, which allowed further data analysis.

In addition, Venn diagrams were used to count and compare the lists of proteins in each group [29]. We also employed STRING version 12, a database containing information on known and predicted protein interactions, for in-depth analyses of cellular functions and disease mechanisms (https://string-db.org/, accessed on 10 January 2024). In addition, we used Reactome, a database that provides detailed representations of biological pathways, including metabolic pathways, signal transduction, and other cellular processes relevant to human biology. We also employed MetaboAnalyst (https://www.metaboanalyst.ca, accessed on 15 January 2024), a web-based platform designed for comprehensive omics data analysis and interpretation. This platform was used to compare serum proteome profiles between non-diabetes and prediabetes/diabetes. The results are presented as a volcano plot, highlighting upregulated and downregulated proteins.

### 2.5. Statistical Analysis

Statistical analyses were conducted using IBM SPSS version 23 (IBM Corp, Armonk, NY, USA). Continuous and categorical variables are reported as mean ± standard deviation and frequency (%), respectively. The normality of each variable was assessed using the Shapiro–Wilk test. Serum zinc and hsCRP levels were log-transformed and expressed as geometric means with standard deviations. To compare anthropometric measurements, metabolic profiles, and other variables between groups, an unpaired Student’s *t*-test was used with *p* < 0.05 being considered to indicate significance. For genotyping analysis, we used SNPStats (https://www.snpstats.net/start.htm, accessed on 3 January 2024) for descriptive analysis, including genotype and allele frequencies with a test for Hardy–Weinberg equilibrium. Furthermore, associations were analyzed based on linear or logistic regression according to the response variable (quantitative or binary disease status, respectively).

## 3. Results

### 3.1. Analyses of Demographic, Clinical, and Biochemical Characteristics of Study Groups

In this study, we evaluated two groups: those without diabetes and those with prediabetes/diabetes. The demographic characteristics and biochemical parameters of the groups are summarized in Table 1. The mean age and sex ratio were similar between the two groups. However, we observed significantly higher values for BMI, WC, and waist-to-hip ratio in the prediabetic/diabetic group than in the nondiabetic group. The mean FBG level was significantly higher in the prediabetic/diabetic group (119.68 ± 8.31 mg/dL) than in the nondiabetic group (87.86 ± 8.08 mg/dL), while the same trend was seen for HbA1C level, which was 6.71 ± 0.79% in the prediabetic/diabetic group and 5.38 ± 0.23% in the nondiabetic group (*p* < 0.05). However, levels of HDL-C, LDL-C, and TG showed significant differences between the groups. Moreover, the inflammatory marker hsCRP was elevated in the prediabetic/diabetic group (2.35 ± 1.13 mg/L) compared with that in the nondiabetic group (1.06 ± 0.89 mg/L, *p* < 0.05). Additionally, plasma Hcy levels were also higher in the prediabetic/diabetic group. Moreover, serum zinc levels were lower in the prediabetic/diabetic group than in the nondiabetic group.

### 3.2. Associations Between SLC30A8 SNPs, HbA1C Level, Metabolic Syndrome, and Serum Zinc Tertiles in Nondiabetic and Prediabetic/Diabetic Groups

The results of the analysis of the SLC30A8 rs13266634 polymorphism and its association with HbA1C levels and serum zinc (grouped into tertiles) for both study groups are presented in Table 2 and Table 3. The genotype distribution was as follows: in the nondiabetic group, 31% were TT, 46% were TC, and 23% were CC; while in the prediabetic/diabetic group, 37% were TT, 45% were TC, and 16% were CC. The allele frequencies were T: 0.54 and C: 0.46 in the nondiabetic group, and T: 0.60 and C: 0.40 in the prediabetic/diabetic group. Using these data, this SNP was determined to be in Hardy–Weinberg equilibrium (*p* > 0.05).

We conducted a further analysis of the association between the genotype at this SNP and HbA1C levels in a sample of 265 individuals. The results indicated that the mean HbA1C levels for the CC genotype in the codominant and recessive models, as well as for the T/C genotype in the overdominant model, were significantly higher than those of the other genotypes. Additionally, an interaction analysis of frequencies of the different genotypes among serum zinc tertiles revealed that the CC genotype in the first tertile of zinc showed a greater mean difference compared with the T/T and T/C genotypes, with an interaction *p*-value of 0.0048, as shown in Table 2.

We also analyzed potential trends linking prediabetes/diabetes with metabolic syndrome. Specifically, we conducted additional analyses of the associations of genotypes at the SLC30A8 SNP with metabolic syndrome and with tertiles of serum zinc levels, as presented in Table 3. The results indicated significant ORs for the risk of metabolic syndrome for the CC genotype in both codominant and recessive models, with values of 4.21 (95% CI: 1.39–12.78, *p* = 0.0056) and 5.00 (95% CI: 1.82–13.71, *p* = 0.0016), respectively. Furthermore, our interaction analysis of this SNP with the tertiles of serum zinc levels and its association with metabolic syndrome (*n* = 265), adjusted for prediabetes/diabetes status, HbA1C, TC, TG, HDL-C, LDL-C, FBG, Hcy, hsCRP, and serum zinc, revealed an OR for the risk of metabolic syndrome of 18.92 (95% CI: 2.47–145.08, *p* = 0.0052) for the first tertile of serum zinc (interaction *p*-value = 0.0052).

### 3.3. Proteomic Profiles Related to Diabetes and Metabolic Syndrome

**Figure 1:**
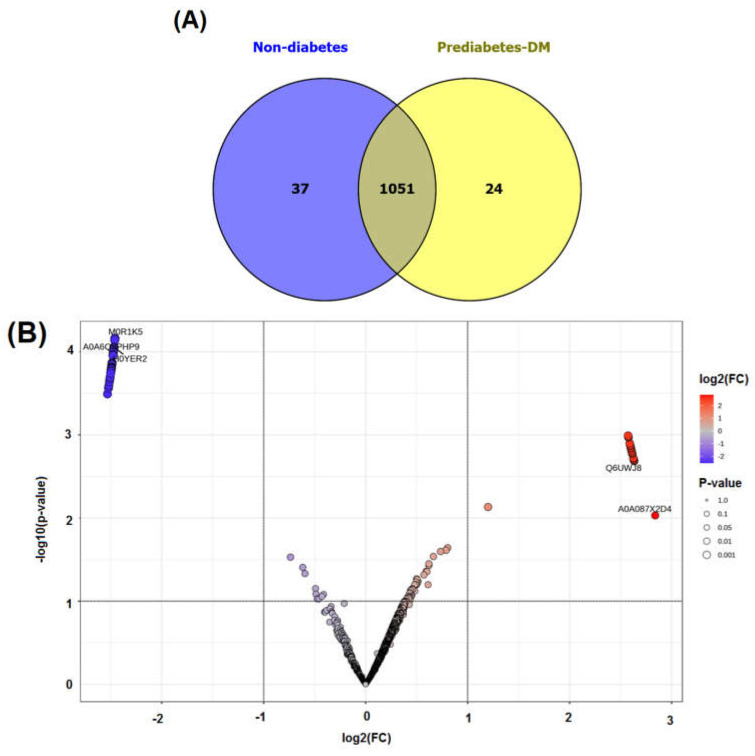
Venn diagram of identified proteins (**A**) and volcano plot of upregulated and downregulated proteins (**B**) in non-diabetes compared with prediabetes/diabetes groups.

A total of 1112 proteins were identified in all of the subjects using proteomic profiling, of which 37 and 24 were expressed exclusively in the nondiabetic and prediabetic/diabetic groups, respectively (Figure 1A). The proteins found exclusively in the nondiabetic group included CREB-regulated transcription coactivator 2, cytoplasmic 60S subunit biogenesis factor ZNF622, and various forms of zinc finger proteins. In contrast, the proteins identified exclusively in the prediabetic/diabetic group included aldehyde dehydrogenase 3 family member B1, trafficking protein particle complex subunit 9, cGMP-dependent protein kinase, and fibroblast growth factor (Supplementary Table S1).

**Figure 2:**
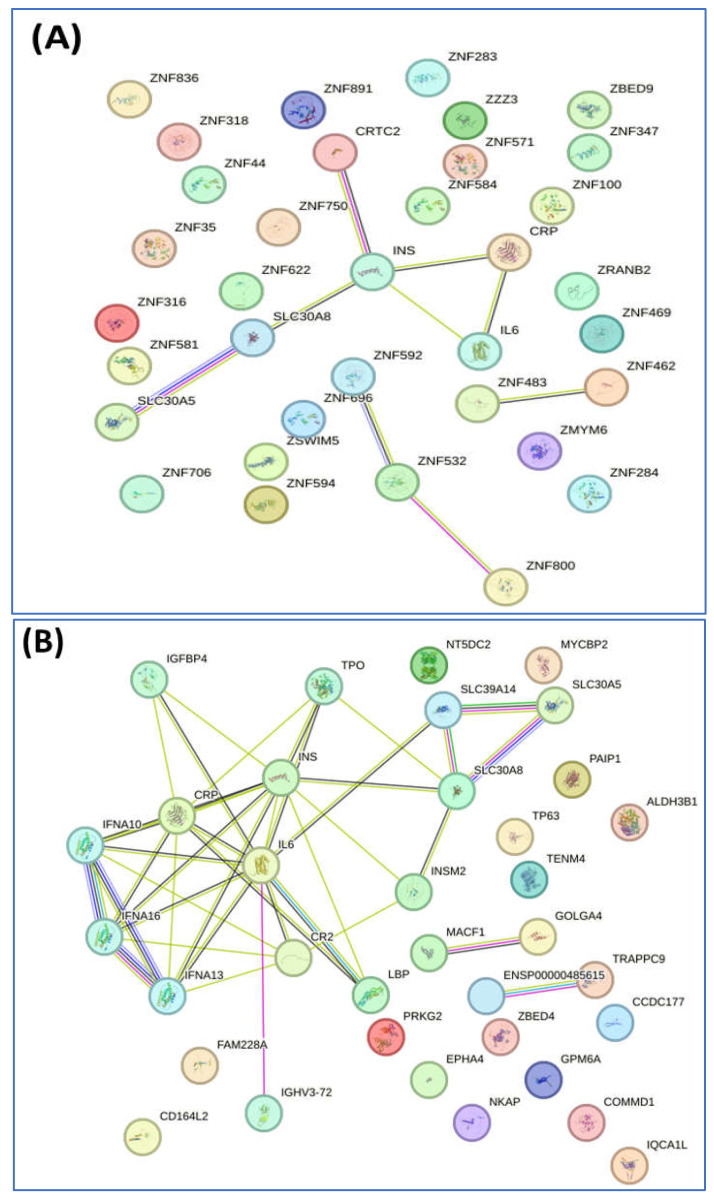

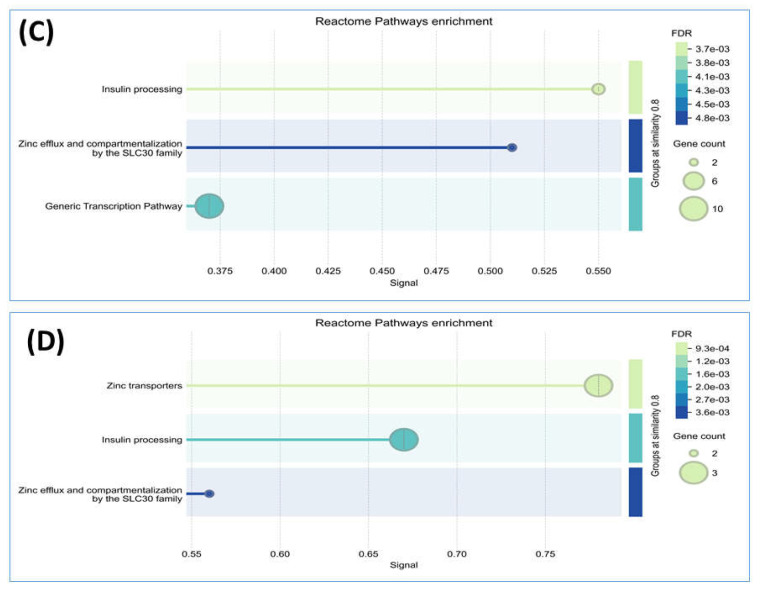
Interaction of unique proteins (network nodes or colored circles represent proteins, and colored lines between the proteins indicate the various types of interaction evidence) and Reactome pathways in the non-diabetes group (**A**,**C**) and prediabetes/diabetes group (**B**,**D**). Node colors represent different levels of interaction, while edge colors denote known, predicted, and other interactions. Colored nodes indicate the query proteins and their first shell of interactors. White nodes represent the second shell of interactors, and empty nodes indicate proteins with unknown 3D structures. Filled nodes show proteins with known or predicted 3D structures. Edges illustrate both functional and physical protein associations, with line colors indicating the type of interaction evidence and line thickness reflecting the strength of the data support.

We conducted a detailed analysis of the proteins exclusively expressed in each group using STRING software version 12.0, which revealed valuable insights. Analysis by this software generally involved inputting a list of 34 unique proteins from the non-diabetes group and 24 unique proteins from the prediabetes/diabetes group, from which the software then generated interaction networks, visualized protein–protein interaction (PPI) patterns (such as interaction between insulin and SLC30A8), and identified functional associations or clusters among the proteins. The results are illustrated in Figure 2A,B. In addition, this approach enhances understanding of the molecular pathways, functional enrichment, and potential biomarkers relevant to our study, as shown by the Reactome pathways. The findings highlighted notable similarities between both groups, particularly in insulin processing and the roles of the SLC30 family in zinc efflux and compartmentalization. Furthermore, our findings showed that the enriched pathway associated with generic transcription was identified in the nondiabetic group, while the zinc transporter pathway was identified in the prediabetes/diabetes groups. This suggests potential avenues for further investigation and understanding of these pathways in relation to diabetes (Figure 2C,D).

We also performed an analysis using MetaboAnalyst software version 5.0, which revealed a total of 40 proteins exhibiting increased expression (upregulated) and 29 proteins showing decreased expression (downregulated) in the prediabetic/diabetic group when compared with the level in the nondiabetic group (Table 4). The findings were visually represented through a volcano plot, which showed the log2-fold changes in the proteins in relation to their statistical significance, with a *p*-value threshold of less than 0.001 (see Figure 1B and Table 4 for details). The proteins upregulated in the prediabetic/diabetic group included zinc finger proteins, tryptophan hydroxylase 1, metallothionein-4 (MT-4), sirtuin1, and glutathione synthetase. Meanwhile, the proteins downregulated in the prediabetic/diabetic group included insulin-like growth factor 1 receptor, T-complex protein 1 subunit gamma, lipopolysaccharide-induced TNF factor, and mitogen-activated protein kinase kinase.

**Figure 3:**
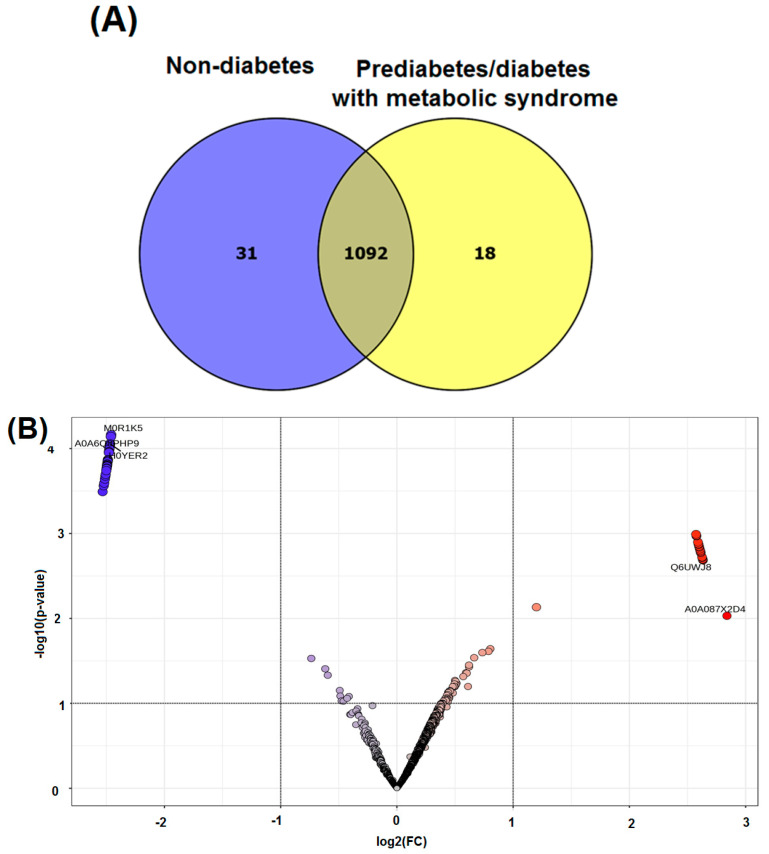
Venn diagram of identified proteins (**A**) and volcano plot of upregulated and downregulated proteins (**B**) in non-diabetes compared with prediabetes/diabetes with metabolic syndrome groups.

In this study, we also employed proteomic analysis to explore the pathways through which zinc influences metabolic processes, revealing potential biomarkers and molecular mechanisms linked to diabetes and metabolic syndrome. A Venn diagram, as presented in Figure 3, was used to visually represent the data, showing 31 proteins exclusively expressed in the nondiabetic group and 18 proteins exclusively expressed in the prediabetic/diabetic with metabolic syndrome group. The analysis revealed that the proteins exclusively expressed in either group were associated with differing metabolic states. Specifically, in the nondiabetic group, the unique proteins identified included ACACB protein, actin-related protein 6, activating signal cointegrator 1 complex subunit 3, and acid phosphatase (ACPP). Conversely, the prediabetic/diabetic group exhibited unique proteins such as aldehyde dehydrogenase 3 family member B1, sialomucin-like 2 protein, cellular tumor antigen p53, and cGMP-dependent protein kinase. These findings provide potential insights into the molecular mechanisms underlying prediabetes/diabetes with metabolic syndrome (Supplementary Table S2).

**Figure 4:**
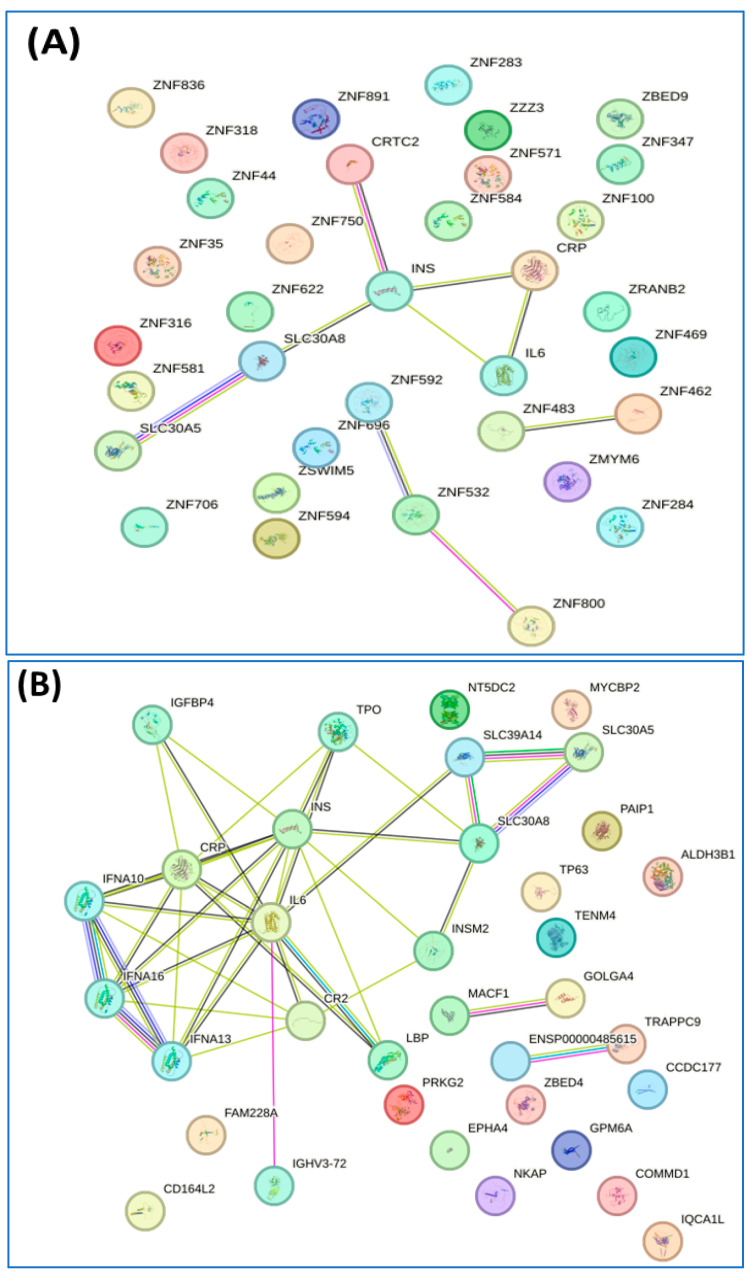

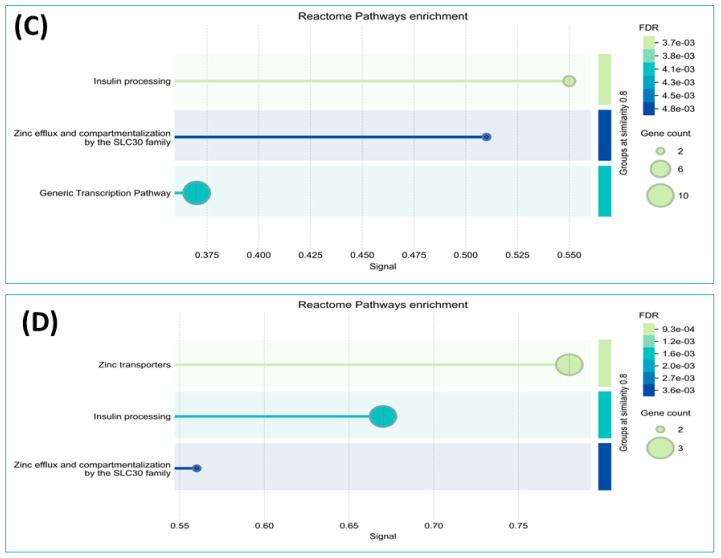
Interaction of unique proteins (network nodes or colored circles represent proteins, and colored lines between the proteins indicate the various types of interaction evidence) and Reactome pathways in non-diabetes group (**A**,**C**) and prediabetes/diabetes with metabolic syndrome group (**B**,**D**). Node colors represent different levels of interaction, while edge colors denote known, predicted, and other interactions. Colored nodes indicate the query proteins and their first shell of interactors. White nodes represent the second shell of interactors, and empty nodes indicate proteins with unknown 3D structures. Filled nodes show proteins with known or predicted 3D structures. Edges illustrate both functional and physical protein associations, with line colors indicating the type of interaction evidence and line thickness reflecting the strength of the data support.

The interactions of the proteins exclusively expressed in each group were then analyzed using STRING software version 12.0. This led to the identification of the pathways of inositol phosphate metabolism and ALKBH3-mediated reversal of alkylation damage in the nondiabetic group, along with the pathway of insulin processing and the roles of the SLC30 family in zinc efflux and compartmentalization in the prediabetic/diabetic group (Figure 4A–D).

The volcano plot indicated a total of 19 upregulated proteins and 31 downregulated proteins when comparing the nondiabetic group with the prediabetic/diabetic group with metabolic syndrome (Figure 3B). Among the upregulated proteins were aldehyde dehydrogenase 3 family member B1, sialomucin-like 2 protein, cGMP-dependent protein kinase, and copper metabolism domain containing 1. We also identified several downregulated proteins, including NOP2/Sun RNA methyltransferase 4, phospholipase C epsilon 1, activating signal cointegrator 1 complex subunit 1, and acyl-CoA synthetase medium chain family member 5 (Table 5).

## 4. Discussion

Prediabetes in older adults is a significant public health concern. The causes of aging-related abnormalities in glucose metabolism are complex and involve various genetic and environmental factors. Interactions between genes and nutrients, between genes and environmental factors, or between different genes may contribute to the pathology of this condition [30]. In particular, studies of gene–nutrient interactions have revealed how specific genetic variants influence metabolism in response to dietary factors, thus affecting the risk of developing prediabetes [30].

In this study, prediabetic/diabetic participants showed significantly greater BMI, WC, and waist-to-hip ratio than those without diabetes. It is well known that increased adiposity, especially visceral fat, is associated with insulin resistance, leading to abnormal glucose metabolism. In addition, dyslipidemia was found in the prediabetic/diabetic group, which is similar to the findings in a previous study in which the prevalence rates of high TC, high TG, high LDL-C, low HDL-C, and dyslipidemia were increased in association with prediabetes and diabetes among middle-aged and older populations in China [31]. Hyperglycemia is linked to elevated levels of hsCRP, as demonstrated in this study. The increase in hsCRP can be attributed to several underlying mechanisms, including insulin resistance, which triggers inflammatory processes, and the presence of excess adipose tissue (especially visceral fat), which releases proinflammatory cytokines [32]. This chronic low-grade inflammation can lead to additional metabolic dysfunction and heighten the risk of developing T2DM. Hyperhomocysteinemia, defined by plasma Hcy levels of 15 μmol/L or higher, is a common finding in prediabetic/diabetic individuals. This aligns with previous research demonstrating that patients with impaired glucose tolerance consistently exhibit elevated Hcy levels compared with those with normal glucose tolerance [33]. This relationship is likely attributable to insulin’s role in reducing the activity of critical enzymes involved in the remethylation and transsulfuration pathways [34]. Furthermore, evidence shows that Hcy thiolactone significantly disrupts insulin signaling by increasing oxidative stress [35].

Our findings showed that serum zinc levels differed between the nondiabetic and prediabetic/diabetic groups, similar to the findings in previous reports [3,4,5,6]. A possible explanation for this association relates to the role of zinc in various glucose metabolic processes, including regulating oxidative stress, insulin synthesis, insulin resistance, and β-cell-related signaling pathways [7,8,9]. Additionally, our study on the interactions between SLC30A8 and serum zinc concentrations for impaired glucose regulation and T2DM produced significant findings that are consistent with other studies [12,13,14] and have important implications. Specifically, we found that individuals with the CC genotype of SLC30A8 rs13266634 demonstrated higher levels of HbA1C than those with the TT genotype (Table 2). The OR of those with the homozygous genotype CC at SLC30A8 rs13266634 compared with TT was 4.21 (95% CI, 1.39–12.78; codominant model) or 5.00 (95% CI, 1.82–13.71; recessive model) for metabolic syndrome, while a lower serum zinc level also increased the OR for metabolic syndrome (Table 3). These findings provide clear supportive evidence of the involvement of this SNP in the risk of T2DM and metabolic syndrome. This is reasonable given that the SLC30A8 gene encodes a zinc transporter that is essential for insulin secretion and glucose metabolism. The increased risk of developing T2DM due to this functionally relevant SNP occurs because it significantly influences the expression and activity of the protein, potentially impairing pancreatic β-cell function. The C variant is prevalent across diverse populations and clearly demonstrates the vital role of genetic factors in the development of metabolic diseases [12,13,16].

In this study, we reported a group of 37 proteins exclusively expressed in the nondiabetic group that were associated with Reactome pathways including insulin processing, zinc efflux and compartmentalization by the SLC30 family, and the generic transcription pathway. Meanwhile, 24 proteins exclusively expressed in the prediabetic/diabetic group were allocated to pathways related to zinc transporters, insulin processing, and zinc efflux and compartmentalization by the SLC30 family (Figure 1 and Figure 2). Dysregulation of zinc transporter proteins can lead to impaired insulin activity and increased oxidative stress, contributing to the development of insulin resistance and beta-cell dysfunction, both of which are key factors in the etiology of diabetes [36]. Zinc efflux and compartmentalization are significantly influenced by the SLC30 family of zinc transporters, particularly SLC30A1 to SLC30A10, which maintains cellular zinc homeostasis. In diabetic patients, dysregulation of these transporters may contribute to altered zinc levels, affecting insulin signaling and pancreatic β-cell function [37]. Studies have indicated that impaired zinc metabolism can exacerbate oxidative stress and inflammation, both of which are central to the pathophysiology of diabetes [3,4,5,19]. Understanding the role of SLC30 transporters in zinc homeostasis offers potential avenues for therapeutic interventions aimed at improving the metabolic health of diabetic patients. Tryptophan hydroxylase 1 was found in the nondiabetic group in this study (Table 4 and Table 5), while a previous study in a rat model reported that this protein was upregulated in rat islets exposed to high glucose and is a regulator of β-cell growth and function [38]. Another of these proteins, CREB-regulated transcription coactivator 2 (CRTC2), plays a significant role in glucose metabolism and insulin signaling by regulating the expression of genes involved in gluconeogenesis and insulin sensitivity, making it an important factor in the pathophysiology of diabetes [39]. A further protein is MT-4, an isoform of metallothionein and a cysteine-rich protein, which acts as a regulator of the homeostasis of metals such as zinc and copper in tissues and functions as a potent antioxidant to protect cells and tissues from oxidative stress, together with emerging pathophysiological roles in pancreatic beta cells [40]. A previous study showed that zinc-induced or genetically enhanced pancreatic MT synthesis prevented diabetes induced by chemicals such as streptozotocin and alloxan, and that zinc pretreatment also prevented spontaneously developed diabetes [41].

Among the proteins found to be significantly highly differentially expressed in the prediabetic/diabetic group, aldehyde dehydrogenase 3 family member B1 (ALDH3B1) is an enzyme potentially involved in the defense against oxidative stress, particularly against lipid peroxidation-derived aldehydes [42]. The increased expression levels of ALDH3B1 in prediabetic/diabetic individuals may reflect the detoxification of aldehydes and the reduction in oxidative stress, both of which are heightened in diabetic conditions. The increased expression of ALDH3B1 was also reported to lead to activation of the NRF2 pathway and reduce the production of intracellular ROS and intracellular lipid peroxidation [43]. Another protein identified in this study, NF- κB-activating protein, is involved in TNF- and IL-1-induced NF-κB activation. This is an important finding, because in the development of insulin resistance and associated metabolic disorders, the NF-κB signaling pathway can be activated, leading to the expression of proinflammatory cytokines that may contribute to the pathogenesis of T2DM [44].

Diabetes and metabolic syndrome are closely linked conditions that share common mechanisms pertaining to insulin resistance, lipid metabolism, and inflammation. Impaired glucose uptake and increased hepatic glucose production also play significant roles in these diseases, creating a vicious cycle that contributes to the progression of both metabolic syndrome and diabetes [45]. We thus further analyzed the profiles of proteins differentially expressed in the groups in this study. Analysis of the interactions of 32 proteins exclusively expressed in the nondiabetic group revealed pathways in which they were particularly enriched, including inositol phosphate metabolism and ALKBH3-mediated reversal of alkylating damage. Meanwhile, the 18 proteins exclusively expressed in the prediabetic/diabetic with metabolic syndrome group were associated with pathways related to insulin processing and zinc efflux and compartmentalization by the SLC30 family (Figure 4). These findings are particularly notable for the following reasons. Inositol phosphate metabolism plays a crucial role in regulating glucose metabolism through various signaling pathways. Inositol phosphates, particularly inositol trisphosphate and inositol hexakisphosphate, act as secondary messengers that influence insulin signaling, glycogen synthesis, and glucose uptake in tissues such as muscle and fat [46]. With regard to the relevance of ALKBH3 in the context of this study, it plays a role in the DNA repair system and thus may be involved in the regulation of glucose metabolism, as the proper functioning of this repair system could protect metabolic organ functions against tissue inflammation, cell death, or senescence [47]. In terms of identifying the category of zinc efflux and compartmentalization, these are crucial processes mediated by the SLC30 family of transporters, which play a significant role in maintaining cellular zinc homeostasis. Dysregulation of zinc levels or abnormal zinc transport can influence insulin signaling, oxidative stress, and inflammation, all of which are key factors in the pathogenesis of metabolic syndrome [48].

cGMP-dependent protein kinase, which was identified in our findings, plays a significant role in vascular function and insulin signaling, highlighting its relevance in the context of diabetes and metabolic syndrome. Specifically, alterations in cGMP signaling can influence insulin sensitivity and glucose metabolism, potentially contributing to the pathophysiology of these conditions [49]. In addition, adenosylhomocysteinease 3 is an enzyme involved in the metabolism of S-adenosylhomocysteine, which may be involved in the regulation of Hcy levels, oxidative stress, and inflammation. In this way, it could contribute to the development and progression of diabetic complications through mechanisms such as disturbed lipid metabolism and impaired nitric oxide production, ultimately exacerbating insulin resistance and metabolic syndrome [50]. Another protein identified in this study, golgin A4, is a protein associated with the Golgi apparatus that is involved in maintaining its structure and function. In the context of diabetes, this protein may play a role in insulin secretion and the regulation of glucose metabolism, and changes in its expression or function could impact the vesicular transport mechanisms necessary for insulin release from pancreatic β cells, potentially contributing to the pathophysiology of diabetes [51]. Furthermore, we found adhesion G-protein-coupled receptor L2 (ADGRL2) in this study; this is a member of the G-protein-coupled receptors that may function in both cell adhesion and signal transduction. In the context of diabetes, ADGRL2 may influence insulin signaling pathways, adipogenesis, and inflammation, which are critical in glucose homeostasis. Mechanistically, ADGRL2 can modulate the activation of G-protein signaling and interact with different ligands, affecting cellular responses that affect insulin sensitivity and energy metabolism [52]. The dysregulation of ADGRL2 signaling could contribute to insulin resistance and the development of T2DM [52]. Most of the proteins found in our study are partly involved in the metabolisms of glucose and insulin, contributing to the etiology of diabetes.

Mechanistically, alterations in these proteins can indicate changes in insulin signaling, glucose metabolism, and metabolic syndrome. Surveying these differences can not only deepen our understanding of the pathophysiology of diabetes but also facilitate early diagnosis, the monitoring of disease progression, and the development of targeted therapies aimed at the molecular mechanisms underlying this disease. This can, in turn, improve patient outcomes and management strategies. However, as data are collected at a single point in time, cross-sectional studies are limited in establishing causality and temporal relationships. Furthermore, such studies may be susceptible to selection bias and confounding factors that could significantly influence the outcomes. To address these limitations, future studies should use a long-term approach to investigate changes over time. The inclusion of larger and more diverse groups of people within the studied sample would also make the findings more generalizable. Furthermore, for future studies, we plan to analyze the protein profiles in relation to different genotypes of the target SNPs to gain a more comprehensive understanding of the relationships between genetics, protein expression, and metabolic conditions.

## 5. Conclusions

In conclusion, this study has shown that the rs13266634 genetic polymorphism of SLC30A8 is associated with the development of T2DM, particularly in older adults, through mechanisms involving beta-cell dysfunction and insulin secretion dysregulation. Variants of SLC30A8 can affect the transport and availability of zinc, an essential trace element vital for insulin crystallization into its hexameric form. Serum zinc concentrations can serve as a biomarker of the risk of diabetes, with low levels potentially contributing to enhanced oxidative stress and inflammation, both of which are recognized as factors involved in insulin resistance and β-cell dysfunction. The profiles of proteins differentially expressed in the blood serum of nondiabetic and diabetic individuals can reveal distinct biomarkers associated with the metabolic dysfunction, inflammation, and oxidative stress observed in diabetes. 

## Figures and Tables

**Table 1:** General characteristics and biochemical parameters of the study population.

Characteristics	Total (*n* = 265)	Non-Diabetes Group (*n* = 116)	Prediabetes/Diabetes Group (*n* = 149)
Age (years)	67.38 ± 3.88	67.21 ± 3.47	67.52 ± 4.18
Male (*n*, %)	183 (69.1%)	78 (67.2%)	105 (70.5%)
BMI (kg/m^2^)	23.45 ± 3.68	22.37 ± 3.40	24.28 ± 3.69 ^a^
Waist circumference (cm)	85.33 ± 10.55	82.19 ± 9.73	87.69 ± 10.56 ^a^
Waist–hip ratio	94.36 ± 7.26	92.10 ± 6.68	96.08 ± 7.22 ^a^
Systolic blood pressure (mmHg)	132.77 ± 20.92	132.40 ± 23.52	133.05 ± 18.74
Diastolic blood pressure (mmHg)	78.51 ± 11.65	76.94 ± 11.53	79.73 ± 11.64
Fasting plasma glucose (mg/dL)	93.65 ± 22.37	87.86 ± 8.08	119.68 ± 8.31 ^a^
HbA1C (%)	5.92 ± 1.03	5.38 ± 0.23	6.71 ± 0.79 ^a^
Triglyceride (mg/dL)	117.04 ± 76.00	111.37 ± 95.39	158.25 ± 47.59 ^a^
Total cholesterol (mg/dL)	229.26 ± 43.08	224.16 ± 43.53	233.24 ± 42.45
Low-density lipoprotein cholesterol (mg/dL)	129.37 ± 32.68	123.25 ± 31.21	135.18 ± 38.77 ^a^
High-density lipoprotein cholesterol (mg/dL)	64.13 ± 17.41	66.97 ± 18.82	61.93 ± 15.95 ^a^
Albumin (g/dL)	4.51 ± 0.25	4.49 ± 0.25	4.53 ± 0.24
Alanine transaminase (U/L)	19.73 ± 9.78	18.80 ± 8.55	20.46 ± 10.60
Aspartate transaminase (U/L)	22.799 ± 8.92	22.47 ± 7.22	23.04 ± 10.07
Creatinine (mg/dL)	0.89 ± 0.16	0.86 ± 0.11	0.92 ± 0.14
Blood urea nitrogen (mg/dL)	13.09 ± 2.78	12.92 ± 2.82	13.22 ± 2.74
Uric acid (mg/dL)	5.70 ± 1.30	5.53 ± 1.17	5.84 ± 1.28
hs-C-reactive protein * (mg/L)	1.31 ± 0.96	1.06 ± 0.89	2.35 ± 1.13 ^a^
Homocysteine (mmol/L)	16.89 ± 3.74	13.08 ± 3.64	17.62 ± 3.97 ^a^
Serum zinc * (µg/L)	708.09 ± 126.57	742.03 ± 109.25	602 ± 171.39 ^a^

^a^ Significant difference (*p* < 0.05) from non-diabetes group; * Geometric mean.

**Table 2:** Allele and genotype frequency and association between SLC30A8, HbA1C level, and serum zinc tertiles in non-diabetes and prediabetes/diabetes groups.

SNP1 (SLC30A8 rs13266634) Allele Frequencies									
	**All subjects (*n* = 265)**			**Non-diabetes (*n* = 116)**		**Prediabetes/diabetes (*n* = 149)**			
**Allele**	**Count**		**Proportion**	**Count**	**Proportion**	**Count**	**Proportion**		
T	305		0.58	125	0.54	180	0.6		
C	225		0.42	107	0.46	118	0.4		
**Genotype**									
T/T	91		0.34	36	0.31	55	0.37		
T/C	123		0.46	53	0.46	70	0.47		
C/C	51		0.19	27	0.23	24	0.16		
**SNP1 exact test for Hardy–Weinberg equilibrium (*n* = 265)**									
	**N11**		**N12**	**N22**	**N1**	**N2**	***p*** **-** **value**		
All subjects	91		123	51	305	225	0.45		
Non-diabetes	36		53	27	125	107	0.45		
Prediabetes/diabetes	55		70	24	180	118	0.86		
**SNP1 association with HbA1C (*n* = 265, adjusted by group)**									
**Model**	**Genotype**		***n***	**Response mean (s.e.)**	**Difference (95% CI)**	***p*** **-** **value**	**AIC**		**BIC**
Codominant	T/T		91	5.85 (0.07)	0.00	< 0.0001	583.2		601.1
	T/C		123	5.7 (0.03)	−0.12 (−0.31–0.08)				
	C/C		51	6.3 (0.23)	0.57 (0.32–0.81)				
Dominant	T/T		91	5.85 (0.07)	0.00	0.42	612.3		626.6
	T/C-C/C		174	5.88 (0.07)	0.08 (−0.11–0.27)				
Recessive	T/T-T/C		214	5.77 (0.03)	0.00	< 0.0001	582.6		596.9
	C/C		51	6.3 (0.23)	0.63 (0.41–0.86)				
Overdominant	T/T-C/C		142	6.01 (0.09)	0.00	0.0005	600.8		615.2
	T/C		123	5.7 (0.03)	−0.32 (−0.50–−0.14)				
**Interaction analysis with covariate serum zinc tertiles (*n* = 265)**									
	**T1**			**T2**			**T3**		
	***n***	**HbA1C** **mean (s.e.)**	**Difference (95% CI)**	***n***	**HbA1C** **mean (s.e.)**	**Difference (95% CI)**	***n***	**HbA1C** **mean (s.e.)**	**Difference (95% CI)**
T/T	23	5.95 (0.14)	0.00	34	5.76 (0.07)	−0.11 (−0.48–0.26)	34	5.89 (0.14)	−0.03 (−0.40–0.34)
T/C	42	5.8 (0.06)	−0.14 (−0.49–0.21)	40	5.65 (0.05)	−0.19 (−0.55–0.17)	41	5.66 (0.05)	−0.19 (−0.55–0.17)
C/C	23	6.92 (0.47)	1.01 (0.61–1.41)	15	5.94 (0.16)	0.09 (−0.36–0.55)	13	5.62 (0.13)	0.09 (−0.39–0.57)
Interaction *p*-value: 0.0048									

**Table 3:** Association and interaction analysis of SLC30A8 with metabolic syndrome and tertiles of serum zinc.

SNP Association with Metabolic Syndrome (*n* = 265), Adjusted by Prediabetes/Diabetes Status, HbA1C, TC, TG, HDL, LDL, FBG, Hcy, hsCRP, Serum Zinc, and Tertile of Serum Zinc									
Model	Genotype		No MS	MS	OR (95% CI)		*p*-Value	AIC	BIC
Codominant	T/T		66 (33%)	25 (38.5%)	1.00		0.0056	214.4	268.1
	T/C		103 (51.5%)	20 (30.8%)	0.73 (0.30–1.79)				
	C/C		31 (15.5%)	20 (30.8%)	4.21 (1.39–12.78)				
Dominant	T/T		66 (33%)	25 (38.5%)	1.00		0.62	222.5	272.7
	T/C-C/C		134 (67%)	40 (61.5%)	1.22 (0.55–2.72)				
Recessive	T/T-T/C		169 (84.5%)	45 (69.2%)	1.00		0.0016	212.9	263
	C/C		31 (15.5%)	20 (30.8%)	5.00 (1.82–13.71)				
Overdominant	T/T-C/C		97 (48.5%)	45 (69.2%)	1.00		0.055	219.1	269.2
	T/C		103 (51.5%)	20 (30.8%)	0.46 (0.21–1.03)				
**Interaction analysis of SNP with tertiles of serum zinc level in association with metabolic syndrome (*n* = 265), adjusted by prediabetes/diabetes status, HbA1C, TC, TG, HDL, LDL, FBG, Hcy, hsCRP, and serum zinc)**									
	**Tertile 1 of serum zinc**			**Tertile 2 of serum zinc**			**Tertile 3 of serum zinc**		
	**No Ms**	**MS**	**OR** **(95% CI)**	**No MS**	**MS**	**OR** **(95% CI)**	**No MS**	**MS**	**OR** **(95% CI)**
T/T	16	7	1.00	25	9	6.58 (0.99–43.67)	25	9	1.46 (0.16–13.18)
T/C	30	12	4.51 (0.85–23.91)	39	1	0.18 (0.01–3.06)	34	7	2.03 (0.21–19.87)
C/C	14	9	18.92 (2.47–145.08)	10	5	5.12 (0.56–47.23)	10	3	4.52 (0.33–62.35)
Interaction *p*-value: 0.0052									

**Table 4:** Upregulated and downregulated proteins compared between non-diabetes and prediabetes/diabetes group from the volcano plot (*p* < 0.001).

	↑ Upregulated Proteins (*n* = 40)			↓ Downregulated Proteins (*n* = 29)		
	Protein ID	Protein Name	log2 (FC)	Protein ID	Protein Name	log2 (FC)
1	Q6ZNA1	Zinc finger protein 836	2.522	P08069	Insulin-like growth factor 1 receptor	−1.126
2	A0A1U9X8W9	ZBED9	2.506	B4DUR8	T-complex protein 1 subunit gamma	−1.209
3	Q5VUA4	Zinc finger protein 318	2.506	A0A0G2JLC4	Lipopolysaccharide-induced TNF factor	−1.296
4	Q9P0T4	Zinc finger protein 581	2.505	C7DUW4	mitogen-activated protein kinase kinase	−1.337
5	Q96JF6	Zinc finger protein 594	2.505	A0A087WWY0	deleted	−1.406
6	D3DSZ2	deleted	2.505	A0A087X2D4	Aldehyde dehydrogenase 3 family member B1	−2.573
7	A0A494C1V2	Zinc finger protein 891	2.502	Q9NQR7	Coiled coil domain-containing protein 177	−2.580
8	K7EQN0	Zinc finger protein 532	2.502	Q6UWJ8	CD164 sialomucin-like 2 protein	−2.581
9	Q9P217	Zinc finger SWIM domain-containing protein 5	2.501	H7C169	Copper metabolism domain containing 1	−2.585
10	O95218	Zinc finger Ran-binding domain-containing protein 2	2.497	Q5SR47	Complement C3d receptor 2	−2.585
11	A0A0G2JMF9	Zinc finger protein 705G	2.497	F5GZZ5	Receptor protein-tyrosine kinase	−2.590
12	C9J283	Zinc finger ZZ-type containing 3	2.497	H7C4B8	Family with sequence similarity 228 member A	−2.591
13	K7ELU5	Zinc finger protein 571	2.496	A0A7U3JVZ5	Fibroblast growth factor	−2.592
14	X6RCN5	Zinc finger MYM-type containing 6	2.496	C9JHJ5	Golgin A4	−2.594
15	Q5VZN3	Zinc finger protein 483	2.495	D6R9D2	Neuronal membrane glycoprotein M6-a	−2.594
16	P15621	Zinc finger protein 44	2.494	A0A087WVA7	IQ motif containing with AAA domain 1 like	−2.598
17	H3BS19	Zinc finger protein 469	2.493	E9PNZ4	Microtubule actin crosslinking factor 1	−2.601
18	C9JGR2	Zinc finger protein 35	2.493	A0A499FJI4	RCR-type E3 ubiquitin transferase	−2.604
19	M0R2W6	Zinc finger protein 584	2.491	Q8N5F7	NF-kappa-B-activating protein	−2.606
20	A6NEH8	ZNF503-AS2	2.490	A0A024R250	deleted	−2.606
21	Q969S3	Cytoplasmic 60S subunit biogenesis factor ZNF622	2.490	Q9H857	5′-nucleotidase domain-containing protein 2	−2.607
22	Q8IYN0	Zinc finger protein 100	2.490	D6REB4	Poly(A) binding protein interacting protein 1	−2.608
23	M0QZE2	Zinc finger protein 347	2.488	A0A024R930	deleted	−2.612
24	A0A7P0N7C4	Zinc finger protein 142	2.487	A0A140VJM3	cGMP-dependent protein kinase	−2.612
25	H3BLX4	Zinc finger protein 462	2.486	B7ZLP5	SAFB protein	−2.617
26	A0A494C0U8	Zinc finger protein 283	2.483	Q6N022	Teneurin−4 (Ten−4)	−2.634
27	Q32MQ0	Zinc finger protein 750	2.482	C9D7D0	Cellular tumor antigen p53	−2.643
28	B9EH69	ZNF658 protein	2.480	A0A0J9YWK7	Trafficking protein particle complex subunit 9	−2.644
29	B3VRW5	Tryptophan hydroxylase 1	2.477	O75132	Zinc finger BED domain-containing protein 4	−2.737
30	Q2TB10	Zinc finger protein 800	2.477			
31	Q5T4K5	CREB regulated transcription coactivator 2	2.476			
32	Q92610	Zinc finger protein 592	2.474			
33	H0YC70	Zinc finger protein 706	2.470			
34	A6NFI3	Zinc finger protein 316	2.470			
35	Q2VY69	Zinc finger protein 284	2.464			
36	E5RG39	Zinc finger protein 696	2.462			
37	B2RN90	Zinc finger protein 776	2.453			
38	P47944	Metallothionein-4 (MT-4)	1.236			
39	I1Y8W7	Sirtuin 1	1.213			
40	A0A2R8Y7I7	Glutathione synthetase	1.002			

↑ Upregulated proteins and ↓ Downregulated proteins compared with non-diabetes group with *p* < 0.001.

**Table 5:** Upregulated and downregulated proteins compared between non-diabetes and prediabetes/diabetes with metabolic syndrome groups from the volcano plot.

	↑ Upregulated Proteins (*n* = 19)			↓ Downregulated Proteins (*n* = 31)		
	Protein ID	Protein Names	log2 (FC)	Protein ID	Protein Names	log2 (FC)
1	A0A087X2D4	Aldehyde dehydrogenase 3 family member B1	2.839	M0R1K5	NOP2/Sun RNA methyltransferase 4	−2.459
2	Q6UWJ8	CD164 sialomucin-like 2 protein	2.633	A0A6Q8PHP9	Phospholipase C epsilon 1	−2.460
3	C9D7D0	Cellular tumor antigen p53	2.631	H0YER2	Activating signal cointegrator 1 complex subunit 1	−2.468
4	A0A140VJM3	cGMP-dependent protein kinase	2.630	A6N6J7	[histone H3]-trimethyl-L-lysine(4) demethylase	−2.469
5	Q9NQR7	Coiled-coil domain-containing protein 177	2.627	F2Z3J2	Proteasome 26S subunit, non-ATPase 5	−2.471
6	H7C169	Copper metabolism domain-containing 1	2.625	A0A087WTR4	Acyl-CoA synthetase medium chain family member 5	−2.474
7	F5GZZ5	Receptor protein-tyrosine kinase	2.614	E7EVL1	Adenylate cyclase type 8	−2.477
8	A0A7U3JVZ5	Fibroblast growth factor (FGF)	2.612	F8WDK8	Ribosomal protein L22 like 1	−2.477
9	C9JHJ5	Golgin A4	2.610	Q96HN2	Adenosylhomocysteinase 3 (AdoHcyase 3)	−2.477
10	A0A087WVA7	IQ motif containing with AAA domain 1 like	2.606	Q5T0Y8	Sphingomyelin phosphodiesterase acid like 3B	−2.478
11	E9PNZ4	Microtubule actin crosslinking factor 1	2.601	X2CV47	AKT1m transcript variant 3	−2.486
12	D6R9D2	Neuronal membrane glycoprotein M6-a	2.599	Q8NFB6	AID	−2.487
13	D6REB4	Poly(A) binding protein interacting protein 1	2.595	D6RB24	NECAP endocytosis associated 2	−2.488
14	H7C4B8	Family with sequence similarity 228 member A	2.594	Q00722	Phosphoinositide phospholipase C-beta-2)	−2.489
15	A0A499FJI4	RCR-type E3 ubiquitin transferase	2.590	Q9Y573	Actin-binding protein IPP	−2.492
16	Q658V8	Uncharacterized protein DKFZp666C182	2.577	E7EMD6	A-kinase anchoring protein 10	−2.493
17	O94763	Protein phosphatase 1 regulatory subunit 19	2.576	P36896	Activin receptor type-1B	−2.494
18	O75132	Zinc finger BED domain-containing protein 4	2.573	H0Y3V3	Adhesion G protein-coupled receptor L2	−2.494
19	B4DUR8	T-complex protein 1 subunit gamma	1.200	A0A7P0MKV3	Mitochondrial ribosomal protein S22	−2.494
20				A0A1W2PR84	Adhesion G protein-coupled receptor V1	−2.494
21				E5RIU2	ADP ribosylation factor GTPase activating protein 1	−2.494
22				Q9BRH5	Diacylglycerol O-acyltransferase	−2.495
23				A0A2R8YG22	Abhydrolase domain containing 5, lysophosphatidic acid acyltransferase	−2.497
24				P62701	Small ribosomal subunit protein eS4, X isoform	−2.498
25				Q9P212	1-phosphatidylinositol 4,5-bisphosphate phosphodiesterase epsilon-1	−2.500
26				C9JFR9	Cytochrome P450 family 8 subfamily B member 1	−2.506
27				F8WER2	ADP ribosylation factor like GTPase 5A	−2.508
28				Q6LBH1	ACPP (Acid phosphatase)	−2.512
29				J3KNJ4	Activating signal cointegrator 1 complex subunit 3	−2.518
30				F8VRL1	Actin related protein 6	−2.522
31				Q4G170	ACACB protein	−2.532

↑ Upregulated proteins and ↓ Downregulated proteins compared with non-diabetes group with *p* < 0.001.

## Data Availability

The data used to support the findings of this study can be made available by the corresponding author upon request.

## Supplementary Materials

The following supporting information can be downloaded at: https://www.mdpi.com/article/10.3390/jcm14030790/s1. Table S1: Unique proteins in non-diabetes and prediabetes/diabetes groups from the Venn diagram; Table S2: Unique proteins in non-diabetes and prediabetes/diabetes with metabolic syndrome groups from the Venn diagram.
