# Supplementary material for: Genetic Polymorphism of Zinc Transporter-8 Gene (SLC30A8), Serum Zinc Concentrations, and Proteome Profiles Related to Type 2 Diabetes in Elderly
Source: J Clin Med. 2025 Jan 25;14(3):790. doi: 10.3390/jcm14030790 (PMC11818826; doi:10.3390/jcm14030790)
Supplement: Supplementary file 1 [file jcm-14-00790-s001.zip › jcm-3433837-supplementary.pdf]

Supplementary Table S1. Unique proteins in non-diabetes and prediabetes/diabetes groups from the Venn diagram.

Unique proteins in non-diabetes group (N = 37)		Unique proteins in prediabetes/diabetes group (N = 24)	
**Protein ID**	**Protein Name**	**Protein ID**	**Protein Name**
A0A0G2JMF9	Zinc finger protein 705G	A0A087WVA7	IQ motif containing with AAA domain 1 like
A0A1U9X8W9	ZBED9	A0A087X2D4	Aldehyde dehydrogenase 3 family member B1
A0A494C0U8	Zinc finger protein 283	A0A0J9YWK7	Trafficking protein particle complex subunit 9
A0A494C1V2	Zinc finger protein 891	A0A140VJM3	cGMP-dependent protein kinase cGMP-dependent protein kinase
A0A7P0N7C4	Zinc finger protein 142	A0A499FJI4	RCR-type E3 ubiquitin transferase RCR-type E3 ubiquitin transferase
A6NEH8	ZNF503-AS2 ZNF503-AS2	A0A7U3JVZ5	Fibroblast growth factor
A6NFI3	Zinc finger protein 316	B7ZLP5	SAFB protein
B2RN90	Zinc finger protein 776	C9D7D0	Cellular tumor antigen p53
B3VRW5	Tryptophan hydroxylase 1	C9JHJ5	Golgin A4
B9EH69	ZNF658 protein	D6R9D2	Neuronal membrane glycoprotein M6-a
C9J283	Zinc finger ZZ-type containing 3 Zinc finger ZZ-type containing 3	D6REB4	Poly(A) binding protein interacting protein 1 Poly(A) binding protein interacting protein 1
C9JGR2	Zinc finger protein 35	E9PNZ4	Microtubule actin crosslinking factor 1
E5RG39	Zinc finger protein 696	F5GZZ5	Receptor protein-tyrosine kinase Receptor protein-tyrosine kinase
H0YC70	Zinc finger protein 706	H7C169	Copper metabolism domain containing 1
H3BLX4	Zinc finger protein 462	H7C4B8	Family with sequence similarity 228 member A
K7ELU5	Zinc finger protein 571	O75132	Zinc finger BED domain-containing protein 4
K7EQN0	Zinc finger protein 532	Q5SR47	Complement C3d receptor 2
M0QZE2	Zinc finger protein 347	Q6N022	Teneurin-4 Teneurin-4
M0R2W6	Zinc finger protein 584	Q6UWJ8	CD164 sialomucin-like 2 protein CD164 sialomucin-like 2 protein
O95218	Zinc finger Ran-binding domain-containing protein 2 Zinc finger Ran-binding domain-containing	Q8N5F7	NF-kappa-B-activating protein NF-kappa-B-activating protein
P15621	Zinc finger protein 44	Q9H857	5'-nucleotidase domain-containing protein 2 5'-nucleotidase domain-containing protein 2
Q2TB10	Zinc finger protein 800	Q9NQR7	Coiled-coil domain-containing protein 177 Coiled-coil domain-containing protein 177
Q2VY69	Zinc finger protein 284		
Q32MQ0	Zinc finger protein 750		
Q5T4K5	CREB regulated transcription coactivator 2		
Q5VUA4	Zinc finger protein 318		
Q5VZN3	Zinc finger protein 483		
Q6ZNA1	Zinc finger protein 836		

Q8IYN0	Zinc finger protein 100		
Q92610	Zinc finger protein 592		
Q969S3	Cytoplasmic 60S subunit biogenesis factor ZNF622		
Q96JF6	Zinc finger protein 594		
H3BS19	Zinc finger protein 469		
Q9P0T4	Zinc finger protein 581		
Q9P217	Zinc finger SWIM domain-containing protein 5<br>Zinc finger SWIM domain-containing protein 5		

Supplementary Table S2. Unique proteins in non-diabetes and prediabetes/diabetes with metabolic syndrome groups from the Venn diagram.

**Unique proteins in non-diabetes group (N = 31)**		**Unique proteins in prediabetes/diabetes with metabolic syndrome (N = 18)**	
**Protein ID**	**Protein Name**	**Protein ID**	**Protein Name**
Q4G170	ACACB protein	A0A087X2D4	Aldehyde dehydrogenase 3 family member B1
F8VRL1	Actin-related protein 6	Q6UWJ8	CD164 sialomucin-like 2 protein
J3KNJ4	Activating signal cointegrator 1 complex subunit 3	C9D7D0	Cellular tumor antigen p53
Q6LBH1	ACPP (Acid phosphatase)	A0A140VJM3	cGMP-dependent protein kinase
F8WER2	ADP ribosylation factor like GTPase 5A	Q9NQR7	Coiled-coil domain-containing protein 177
C9JFR9	Cytochrome P450 family 8 subfamily B member 1	H7C169	Copper metabolism domain-containing 1
Q9P212	Phospholipase C-epsilon-1 (PLC-epsilon-1)	F5GZZ5	Receptor protein-tyrosine kinase
P62701	Small ribosomal subunit protein eS4, X isoform	A0A7U3JVZ5	Fibroblast growth factor (FGF)
A0A2R8YG22	Abhydrolase domain-containing 5, lysophosphatidic acid acyltransferase	C9JHJ5	Golgin A4
Q9BRH5	Diacylglycerol O-acyltransferase 1	A0A087WVA7	IQ motif containing AAA domain 1 like
E5RIU2	ADP ribosylation factor GTPase activating protein 1	E9PNZ4	Microtubule actin crosslinking factor 1
A0A1W2PR84	Adhesion G protein-coupled receptor V1	D6R9D2	Neuronal membrane glycoprotein M6-a
A0A7P0MKV3	Mitochondrial ribosomal protein S22	D6REB4	Poly(A)-binding protein interacting protein 1
H0Y3V3	Adhesion G protein-coupled receptor L2	H7C4B8	Family with sequence similarity 228 member A
P36896	Serine/threonine-protein kinase receptor R2	A0A499FJI4	RCR-type E3 ubiquitin transferase
E7EMD6	A-kinase anchoring protein 10	Q658V8	Uncharacterized protein DKFZp666C182
Q9Y573	Actin-binding protein IPP	O94763	Protein NNX3
Q00722	Phosphoinositide phospholipase C-beta-2	O75132	Zinc finger BED domain-containing protein 4
D6RB24	NECAP endocytosis-associated 2		
Q8NFB6	AID		
X2CV47	AKT1m transcript variant 3		
Q5T0Y8	Sphingomyelin phosphodiesterase acid like 3B		
Q96HN2	Adenosylhomocysteinase 3 (AdoHcyase 3)		
F8WDK8	Ribosomal protein L22 like 1		
E7EVL1	Adenylate cyclase type 8		
A0A087WTR4	Acyl-CoA synthetase medium chain family member 5		
F2Z3J2	Proteasome 26S subunit, non-ATPase 5		
A6N6J7	[histone H3]-trimethyl-L-lysine (4) demethylase		
H0YER2	Activating signal cointegrator 1 complex subunit 1		
A0A6Q8PHP9	Phospholipase C epsilon 1		
M0R1K5	NOP2/Sun RNA methyltransferase 4		
